# Metagenomic next-generation sequencing for detecting *Aspergillosis* pneumonia in immunocompromised patients: a retrospective study

**DOI:** 10.3389/fcimb.2023.1209724

**Published:** 2023-12-22

**Authors:** Yan Shi, Jin-Min Peng, Xiao-Yun Hu, Qi-Wen Yang, Yao Wang

**Affiliations:** ^1^ Department of Medical ICU, Peking Union Medical College Hospital, Peking Union Medical College and Chinese Academy of Medical Sciences, Beijing, China; ^2^ Department of Clinical Laboratory, Peking Union Medical College Hospital, Peking Union Medical College and Chinese Academy of Medical Sciences, Beijing, China

**Keywords:** metagenomic next-generation sequencing (mNGS), diagnosis, invasive pulmonary *aspergillosis* (IPA), immunocompromised patients (ICP), bronchoalveolar lavage fluid (BALF), conventional microbial tests (CMT)

## Abstract

**Purpose:**

The identification of *Aspergillus* by metagenomic next-generation sequencing (mNGS) remains a challenging task due to the difficulty of nucleic acid extraction. The objective of this study was to determine whether mNGS could provide an accurate and efficient method for detecting invasive pulmonary *aspergillosis* (IPA) in immunocompromised patients (ICP).

**Methods:**

A total of 133 ICP admitted to the ICU between January 2020 and September 2022 were enrolled in the study, of which 46 were diagnosed with IPA and 87 were non-IPA cases. The bronchoalveolar lavage fluid (BALF) was analyzed for the presence of *Aspergillosis* and other co-pathogens using mNGS, and its diagnostic performance was compared to conventional microbial tests (CMTs) that included smear, cultures, serum and BALF galactomannan (GM) test. Clinical composite diagnosis was used as the reference standard

**Results:**

mNGS had a sensitivity, specificity, and accuracy of 82.6%, 97.7%, and 92.5%, respectively, in diagnosing IPA. These findings were comparable to those of the combination of multiple CMTs. Interestingly, the sensitivity of mNGS was superior to that of any single CMT method, as demonstrated by comparisons with smears (8.7%, *P* < 0.001), culture (39.1%, *P* < 0.001), serum GM (23.9%, *P* < 0.001) and BALF GM (69.6%, *P* = 0.031). mNGS was capable of accurately distinguish strains of *Aspergillus* genus, with a consistency of 77.8% with culture. Furthermore, mNGS also identified *A. fumigatus*, *A. flavus*, *A. terrestris*, *A. oryzae* and *Mucor* spp. in culture-negative cases. The sequencing reads of *Aspergillus* by mNGS exhibited extensive variation, ranging from 11 to1702. A positive correlation was observed between the optical density index of BALF GM and unique reads by mNGS (r = 0.607, *P* = 0.001) in BALF-GM positive patients. Notably, mNGS was able to diagnose 35 out of 37 cases with mixed infection, with *P. jirovecii* and *cytomegalovirus* being the most common co-pathogens.

**Conclusions:**

mNGS presents a feasible and remarkably sensitive approach for detecting *Aspergillus* in ICP, thereby serving as a valuable adjunctive tool to CMT. Furthermore, mNGS’s ability to accurately identify fungal species and co-pathogens can assist in guiding appropriate antimicrobial therapy.

## Introduction

In recent years, the prevalence of pulmonary fungal disease has exhibited a significant upward trend due to an increase in high-risk groups requiring immunosuppressive therapy. Invasive pulmonary *aspergillosis* (IPA) is still an important cause of morbidity and mortality (Herbrecht et al., 2002; Gangneux et al., 2010; Schauwvlieghe et al., 2018). However, the non-specific clinical features of IPA, especially in non-neutropaenic patients, often lead to delayed initiation of treatment and higher mortality (Cornillet et al., 2006; Gangneux et al., 2010). Studies have shown that mortality rates can reach up to 90% if the treatment is not administered until 10 days after the first clinical or radiological sign of the disease, but fall to 40% when treatment is administered early (von Eiff et al., 1995). Therefore, timely and accurate diagnosis is of great significance.

Unfortunately, the diagnosis of *Aspergillus* infections poses a significant challenge. While histopathology is often recommended as a ‘gold standard’, obtaining a sufficient quantity of biopsy samples from the infected site can be difficult, resulting in a high false-negative rate (Bialek et al., 2002). Fungal culture is currently the most commonly used diagnostic tool, but is time-consuming and offer a low yield (Perfect et al., 2001; Arvanitis and Mylonakis, 2015). Although smear by microscopy can identify *Aspergillus* hyphae more quickly than culture, it requires a heavy pathogen burden in the lung and experienced microbiologists to ensure the detection, making it insensitive and biased. In the past decade, the galactomannan (GM) test has been widely used in the diagnosis of IPA, but its sensitivity in serum samples from non-neutropenic hosts is limited due to low fungal load in the lesions and a low chance of antigen appearing in the bloodstream (Stergiopoulou et al., 2007; Cordonnier et al., 2009). BALF GM test is superior to serum GM in the diagnosis of IPA, but its results are affected by standardization of the sampling, diverse mycological procedures, antimicrobial treatment before sampling and various other factors, making its role in non-neutropenic patients uncertain (Maertens et al., 2009; Racil et al., 2011; Brownback et al., 2013; Hage et al., 2019). While polymerase chain reaction (PCR) based methods are more rapid, sensitive, and specific, they require specific primers and are not routinely performed in many hospitals. Therefore, there is an urgent need for new technology with a higher sensitivity to improve *Aspergillus* infection diagnosis.

Metagenomic nextgeneration sequencing (mNGS) is considered a promising microbial identification technology, enabling direct pathogen detection without primers or probes. Although mNGS has been widely used in infectious diseases, there is still a scarcity of clinical experience in pulmonary fungal infections. Previous studies have reported mixed results, with some showing that mNGS outperformed conventional microbial tests (CMT) for fungal detection (Li et al., 2018; Miao et al., 2018; Yang et al., 2021), while others found the opposite. (Xie et al., 2019; Fang et al., 2020). It was worth noting that these studies without exception encompassed various fungi, including *Aspergillus*, *Cryptococcus*, *P. jirovecii* and *Candida*, but different techniques may have their advantages in identifying specific fungal species (Miao et al., 2018; Xie et al., 2019; Fang et al., 2020). Identifying filamentous molds such as *Aspergillus* spp. through mNGS remains challenging due to the difficulty of DNA extraction from the thick polysaccharide cell wall, as well as the relatively low fungal load in BALF (Bittinger et al., 2014; Clarke et al., 2018; Han et al., 2019). To date, limited evidence is available, mainly from case reports and small case series (He et al., 2019; Bao et al., 2022; Yang et al., 2022). Our previous study demonstrated the excellent performance of mNGS in identifying pneumonia pathogens in immunocompromised patients (ICP), but subgroup analysis did not reveal its advantage in *Aspergillus* detection due to the small sample size and immature technology (Shi et al., 2022). As an extension of our earlier study, we conducted research in patients with IPA to contribute to this growing field of research by comparing the diagnostic performance between mNGS and CMT, including smear, cultures, serum and BALF GM test.

## Methods

### Study design and participants

This was a retrospective cohort study consisting of patients with suspected pneumonia who were admitted to the medical ICU of Peking Union Medical College Hospital (Beijing, China) between January 2020 and September 2022. Patients were eligible for enrollment if they met all the following criteria: (1) immunocompromised conditions, including but not limited to hematologic malignancies; solid tumors chemotherapeutically treated within the past 28 days; hematopoietic stemcell transplantation or solid organ transplantation; rheumatic diseases; long-term use of corticosteroids (0.3 mg/kg/day of prednisone equivalent for 3 weeks); taken antirheumatic drugs, biological immunomodulators or immunosuppressants (Hill., 2020); (2) undergoing bronchoalveolar lavage; (3) having smear, culture, GM and mNGS results; (4) having detection results for other pathogen, at least including bacterial culture, acid-fast staining and PCR test for *P. jirovecii* and *cytomegalovirus* (CMV). The following exclusion criteria were used: (1) age < 18 years old; (2) mNGS and CMT were not paired (i.e., not conducted simultaneously or on the same day); (3) medical record was incomplete.

This study was approved by the Research Ethics Committee of our hospital. Individual consent for this retrospective analysis was waived.

### Data collection

Demographic and clinical data were extracted from electronic medical records (EMRs), including age, gender, diagnosis of underlying diseases, steroids or immunosuppressants used, severity of illness by the Acute Physiology and Chronic Health Evaluation (APACHE) II and Sequential Organ Failure Assessment (SOFA) score on ICU admission, pulmonary imaging and laboratory findings, empirical antimicrobial therapy, results of CMTs and mNGS, and outcome.

### Criteria of diagnosis of IPA and identification of pathogens

The histopathological findings of hyphae on lung biopsy were considered proven IPA. For probable IPA, the presence of at least one host factor, clinical manifestation and mycologic evidence was required according to the 2020 criteria from the European Organization for Research and Treatment of Cancer/Mycoses Study Group (EORTIC/MSG) (Donnelly et al., 2020). When any of the following thresholds is met, it will be used as mycological evidence. (1) culture and/or histopathological examination positive for *Aspergillus*; (2) a result of GM test was considered positive when optical density index (ODI) values were ≥1.0 in serum or ≥1.0 in BALF or ≥ 0.7 in serum and ≥ 0.8 in BALF. (3) in this study, a modification in diagnostic criteria was the addition of mNGS as one of mycologic testing methods, with at least 50 unique reads from a single species of fungi being considered positive; for pathogens with unique reads less than 50, the diagnosis of pulmonary fungal infection can still be made based on the clinical situation (Wang et al., 2019). In addition, to elucidate the relationship between *Aspergillus* load and its number of unique reads by mNGS, we compared the number of unique reads between culture-positive and culture-negative patients, as well as between GM-positive and GM-negative patients, and also compared the correlation between the ODI value of GM and the number of unique reads.

The final determination of causative pathogens was based on clinical composite diagnostic criteria (i.e., the gold standard), which was made by two senior expert intensivists (YS and JMP) after independently reviewed the EMRs of each patient, and based on clinical symptoms, laboratory findings, chest radiology, microbiologic tests and treatment response. Any disagreement was resolved by in-depth discussion and consensus.

### Procedures of bronchoalveolar lavage and mNGS

BALF was performed by experienced intensivists after local anesthesia with lidocaine in accordance with the standard procedures at our hospital. Briefly, the sampling location was selected based on chest CT images. Three 20-ml fractions of sterile saline were instilled into the target subsegmental bronchi. The extracted BALF was placed in a sterile container and immediately send for mNGS test and CMTs. Microbiological tests were completed by the clinical laboratory of our hospital. Galactomannan enzyme immunoassay (GM-EIA) was performed using PlateliaTM Aspergillus Ag (Bio-Rad), following the manufacturer’s instructions. mNGS used BGISEQ platform for nucleic acid extraction, library construction, high-throughput sequencing, and bioinformatics analysis. The turnaround time was about one working day. Detailed procedures and interpretation of metagenomic data were given in Appendix 1.

### Statistical analyses

We compared the performance of BALF mNGS with the combination of multiple CMTs (including smears, culture, serum GM and BALF GM) and any single CMT for identifying *aspergillosis*. The 2×2 contingency tables were established to determine sensitivity, specificity, positive predictive value (PPV), negative predictive value (NPV) and accuracy. Wilson’s method was used to calculate 95% confidence intervals (CI) for these proportions. The McNemar test was used for comparisons of the diagnostic performance of CMTs and mNGS. The SPSS 22.0 software was used for data analysis, and *P* < 0.05 was considered statistically significant.

## Results

### Patient recruitment and clinical characteristics

During the study period, a total of 196 ICP with suspected pneumonia were admitted to ICU. Of these, 143 patients who underwent both BALF mNGS and CMT were eligible for inclusion. After excluding seven patients with unmatched paired of mNGS and CMTs, two with incomplete medical records and one patient under the age of 18, 133 patients were subjected to final analysis. Based on clinical composite diagnosis, these patients were divided into IPA group (*n* = 46) and non-IPA group (*n* = 87). Due to the lack of histopathological evidence from lung biopsy, all 46 patients were diagnosed with probable IPA.

Demographic and clinical characteristics of both groups were compared and summarized in **Table 1**.The median ages and gender compositions of these two groups were similar, with rheumatic diseases being the most common underlying disease in both groups (IPA group vs. non-IPA group: 58.7% vs. 52.9%, *P* = 0.714). However, hematological malignancies were more prevalent in the IPA group (21.7% vs. 5.7%, *P* = 0.009). Additionally, a greater proportion of IPA patients received cytotoxic or immunosuppressants (50% vs. 33.3%, *P* = 0.042).

**Table 1:** Demographics and clinical characteristics of the study cohort.

Characteristic	IPA group (*n* = 46)	Non IPA group (*n* = 87)	*P* value
Age, years, median (IQR)	53 (48, 65)	57 (51, 67)	0.198
Sex-male, *n* (%)	22 (48)	40 (46)	0.857
Underlying diseases, *n* (%)			
Rheumatic diseases	27 (58.7)	46 (52.9)	0.714
Haematologic malignancy	10 (21.7)	5 (5.7)	0.009
Active solid tumor	4 (8.7)	10 (11.5)	0.770
Solid organ transplantation	1 (2.2)	0 (0)	0.346
Other immunoinflammatory diseases	4 (8.7) ^†^	26 (29.9) ^‡^	0.005
Immunocompromised conditions, *n* (%)			
Prolonged corticosteroid	32 (69.6)	62 (71.3)	0.689
Cytotoxic or immunosuppressants	24 (50.0)	29 (33.3)	0.042
Neutropenia	5 (10.9)	1 (1.1)	0.019
Antimicrobial therapy before sampling, *n* (%)	45 (97.8)	84 (96.6)	1.000
Antibacterial	45 (97.8)	82 (94.3)	0.664
Anti cytomegalovirus	21 (45.7)	39 (44.8)	0.585
Anti *P. jirovecii*	38 (82.6)	58 (66.7)	0.067
Antifungal	12 (26.1)	11 (12.6)	0.058
Disease severity at ICU admission			
APACHE II score, median (IQR)	20 (17, 24)	19 (16, 23)	0.459
SOFA score, median (IQR)	10 (8, 12)	9 (7, 10)	0.433
Invasive mechanical ventilation, *n* (%)	42 (91.3)	74 (85.1)	0.416
Septic shock, *n* (%)	33 (71.7)	28 (32.2)	< 0.001
Laboratory findings at ICU admission, *n* (%)			
WBC (10 ^9^/L), median (IQR)	7.4 (5.3, 10.9)	7.8 (6.0, 11.8)	0.417
Neutrophils (10^9^/L), median (IQR)	5.8 (4.5, 7.7)	6.5 (5.6, 8.5)	0.376
Lymphocytes (10^6/^L), median (IQR)	465 (220, 670)	421 (240, 660)	0.177
CD4 T cell (10^6^/L), median (IQR)	120 (61, 237)	95 (48, 189)	0.078
Chest CT images, *n* (%)			
Double lung lesions	42 (91.3)	80 (92.0)	0.576
Diffuse Interstitial infiltration	40 (87.0)	68 (78.2)	0.251
Multiple patchy or wedge shadowing	28 (60.9)	38 (43.7)	0.059
Multiple nodules	7 (15.2)	5 (5.7)	0.108
Cavitation sign	5 (10.9)	1 (1.1)	0.019
Crescent sign	1 (2.2)	0 (0)	0.346
Halo sign	0 (0)	0 (0)	NaN
Consolidation	21 (45.7)	25 (28.7)	0.058
Pathogens of pneumonia, *n* (%)			
Bacteria	12 (26.1)	22 (38.6)	0.861
Cytomegalovirus	13 (28.3)	27 (47.4)	0.943
*P. jirovecii*	17 (37.0)	33 (57.9)	0.047
*M. tuberculosis*	3 (6.5)	0 (0)	0.086
Influenza virus	3 (6.5)	6 (10.5)	0.728
*Mucor* spp	7 (15.2)	0 (0)	< 0.001
Polymicrobial pneumonia, *n* (%)	37 (80.4)	21 (36.8)	< 0.001
ICU LOS, days, median (IQR)	14.5 (9, 24)	13.0 (8, 25)	0.056
ICU death, *n* (%)	30 (65.2)	40 (46.0)	0.045

APACHE, acute physiology and chronic health evaluation; IPA, invasive pulmonary aspergillosis; LOS, length of stay; SOFA, sequential organ failure assessment.

^†^including nephrotic syndrome (n = 2); idiopathic interstitial lung disease (ILD) (n = 2).

^‡^including nephrotic syndrome (n = 8); ILD (n = 7); myasthenia gravis (n = 2); ulcerative colitis (n = 2); primary immune thrombocytopenia (n = 2); pemphigus (n = 2); goodpastures syndrome (n = 2); and allergic alveolitis (n = 1).

Upon ICU admission, IPA patients were found to a significantly higher proportion of septic shock compared to the control group (71.7% vs.32.2%, *P* < 0.001). Additionally, IPA patients had a higher incidence of neutropenia (10.9% vs. 1.1%, *P* = 0.019) and polymicrobial pneumonia (80.4% vs 36.8%, *P* < 0.001) compared to the control group. The typical radiological features of IPA on chest CT were infrequent, with only 5 cases demonstrating the presence of cavities and 1 case showing the air crescent sign, while none of the patients exhibited the halo sign. In comparison with the non-IPA group, no significant differences were observed in other radiological features between the two groups, except for the higher prevalence of cavities in the IPA group (10.9% vs. 1.1%, *P* = 0.019) (**Table 1**). In addition, 129 (97%) patients were exposed to antimicrobial therapy prior to sample collection, and the types of antimicrobials prescribed were similar between the two groups. Finally, the ICU mortality of IPA patients was higher than that of the control group (65.2% vs. 46.0%, *P* = 0.045).

### Comparison of diagnostic performance between mNGS and CMT methods

Using clinical composite diagnosis as the reference standard, mNGS identified *Aspergillus* specific sequences in 38 of 46 patients with IPA and 2 of 87 patients without IPA, resulting in a sensitivity of 82.6% (95% CI, 68.0%–91.7%), specificity of 97.7% (95% CI, 91.2%–99.6%), PPV of 95.0% (95% CI, 81.8%–99.1%), NPV of 91.4% (95% CI, 83.3%–95.9%) of NPV, and accuracy of 92.5% (95% CI, 83.7%–96.9%). In contrast, the combination of multiple CMTs yielded positive results in 43 of 46 IPA patients and 6 of 87 control patients, corresponding to a sensitivity and specificity of 93.5% (95% CI, 81.1%–98.3%) and 93.1% (95% CI, 85.0%–97.2%), respectively. The PPV, NPV and accuracy were 87.8% (95% CI, 74.5%–94.9%), 96.4% (95%CI, 89.2%–99.1%), and 93.2% (95% CI, 85.3%–98.3%), respectively. These findings were comparable to those of mNGS (all *P > 0.05*).

Furthermore, we evaluated the diagnostic efficacy of a single CMT method. In the IPA group, fungal smear, culture, serum GM, and BALF GM were positive in 4, 18, 11, and 32 cases, respectively, while in the control group, there were 0, 3, 0, and 3 cases, respectively. As shown in **Table 2**, the sensitivity of mNGS was superior to that of any single CMT method, as demonstrated by comparisons with smears (8.7%, *P* < 0.001), culture (39.1%, *P* < 0.001), serum GM (23.9%, *P* < 0.001) and BALF GM (69.6%, *P* = 0.031). The sensitivity of BALF GM was suboptimal, while the sensitivity of serum-GM and culture was similar (P = 0.118), both better than that of smears (P < 0.05).

**Table 2:** Diagnostic performance of mNGS and CMTs for invasive pulmonary aspergillosis.

Methord	Result	IPA group (n)	Non-IPA group(n)	sensitivity% (95% CI)	specificity% (95% CI)	PPV% (95% CI)	NPV% (95% CI)	Accuracy% (95% CI)
mNGS	pos	38	2	82.6 (68.0-91.7)	97.7 (91.2-99.6)	95.0 (81.8-99.1)	91.4 (83.3-95.9)	92.5(83.7–96.9)
	neg	8	85					
Combination of	pos	43	6	93.5 (81.1-98.3)	93.1 (85.0-97.2)	87.8 (74.5-94.9)	96.4 (89.2-99.1)	93.2(85.3–98.3)
multiple CMT	neg	3	81					
Smear	pos	4	0	8.7 (2.8-21.7)^†^	100 (94.7-100)	100 (39.6-100)	67.4(58.6-75.3)^†^	68.4(55.6-78.3)^†^
	neg	42	87					
Culture	pos	18	3	39.1 (25.5-54.6)^†^	96.6 (89.5-99.1)	85.7 (62.6-96.2)	75.0 (65.8-82.5)^†^	76.7(67.3-83.4)^†^
	neg	28	84					
Serum GM	pos	11	0	23.9 (13.1-39.1)^†^	100 (94.7-100)	100 (67.9-100)	71.3 (62.3-78.9)^†^	73.7(63.8-80.3)^†^
	neg	35	87					
BALF GM	pos	32	3	69.6 (54.1-81.8)^† ‡^	96.6 (89.5-99.1)	91.4 (75.8-97.8)	85.7 (6.9-91.7)^†^	87.2(70.7-95.6)
	neg	14	84					

BALF, bronchoalveolar lavage fluid; CMTs, conventional microbiological tests; GM, galactomannan; IPA, invasive pulmonary aspergillosis; mNGS, metagenomic next-generation sequencing; neg, negative; NPV, negative predictive value; pos, positive; PPV, positive predictive value; CI, confidence interval.

^†^The difference was significant between CMT and mNGS based on the McNemar test (P < 0.05).

^‡^The difference was significant between BALFGM and serum GM based on the McNemar test (P < 0.05).

### Consistency comparison and divergent identifications of different methods

**Table 3** presented the comparison of diagnostic consistency among different methods. Of the 43 patients diagnosed with IPA by CMT, only 4 were positive for all four CMT methods and one was positive for three CMT, including culture, BALF and serum GM. Eleven patients were diagnosed by two CMT methods, consisting of 6 with positive culture and BALF-GM, 3 with positive BALF and serum GM, and 2 with positive culture and serum GM. Twenty-four patients (52.2%) were only positive for a single CMT, including 18 with positive BALF-GM, 5 with positive culture and 1 with positive serum GM. The remaining 3 cases were diagnosed by meeting the criteria of serum GM > 0.7 combined with BALF GM > 0.8.

**Table 3:** Consistency comparison of different *Aspergillus* identification methods in 46 IPA patients.

CMT method	Detected by CMT alone	Detected by CMT and mNGS	Detected by mNGS alone
Combination of multiple CMT	8	35	3
Smear	0	4	34
Culture	1	17	21
BALF GM	3	29	9
Serum GM	1	10	28

BALF, bronchoalveolar lavage fluid; CMT, conventional microbial test; GM, galactomannan; IPA, invasive pulmonary aspergillosis; mNGS, metagenomic next-generation sequencing.

mNGS and the combination of multiple CMT methods showed consistency in 35 (76%) IPA patients, while the 8 patients missed by mNGS were diagnosed with BALF GM >1 (*n* = 3, Nos.15, 27 and 40), positive *Aspergillus* culture (*n* = 1, No.37), serum GM >1 (*n* = 1, No.28), and BALF GM > 0.8 combined with serum GM > 0.7 (*n* = 3, Nos.6, 9 and 13), respectively. In comparison, mNGS identified the only mycological evidence in the three cases (Nos. 2, 17 and 20) that were overlooked by CMT. Notably, two patients with hematological malignancies who received antifungal treatment before sampling were among these cases.

### Comparisons of microbiological findings between mNGS and culture in IPA patients

The strains isolated from 18 individuals with positive *aspergillus* culture consisted of *A. fumigatus* (*n* = 13), *A. flavus* (*n* = 7) and *Aspergillus* spp. (*n* = 1). Among these patients, three suffered from multiple *aspergillus* infections (Nos. 21, 29 and 39). Seventeen of the above patients also detected *Aspergillus* through mNGS, with a detection consistency of 77.8%, while three patients showed mismatched results, with *A. flavus* growth in fungal culture and mNGS detecting *A. fumigatus* (No. 5) and *A. terroiris* (Nos. 21 and 39), respectively. Also, mNGS additionally identified *A. flavus* (No.8), *A. terrestris* (Nos.11, 30, and 44), *A. oryzae* (Nos.11, 25 and 44) and *A. niger* (No.41). Furthermore, mNGS analysis for *aspergillus* culture-negative cases showed positive results for *A. fumigatus* (*n* = 17), *A. flavus* (*n* = 2), *A. terrestris* (*n* = 2), and *A. oryzae* (*n* = 1). Notably, mNGS identified *Mucor* spp. infection in seven patients (Nos. 5, 16, 19, 29, 35, 38 and 42), with only one patient (No.29) was identified by culture (**Supplementary Table**).

### Relationship between fungal culture, BALF-GM and *Aspergillus* unique reads by mNGS

Detailed sequencing data of *Aspergillus* from 38 IPA patients identified by mNGS can be found in the Appendix 2. The sequencing reads of *Aspergillus* spp. identified by mNGS exhibited extensive variation (range from 11 to1702). The median reads for different *Aspergillus* genera were as follows: 85 (IQR 45-204) reads for *A. fumigatus*, 131 (IQR 47-206) reads for *A. flavus*, 53 (IQR 41-235) reads for *A. terroirus*, 43 (IQR 31-55) reads for *A. oryzae*, and 23 read for *A. niger* in one case. The unique reads of *mucor* genus was 49 (IQR 43-105). According to the diagnostic threshold of mycological evidence, nine patients (Nos.10, 12, 14, 17, 20, 23, 26 32 and 38) with *Aspergillus* unique reads less than 50 were still diagnosed as IPA by clinical comprehensive analysis, of which six had positive results in at least one CMT method (**Supplementary Table**).

When comparing the relationship between fungal load and sequencing reads, we observed that the

**Figure 1:**
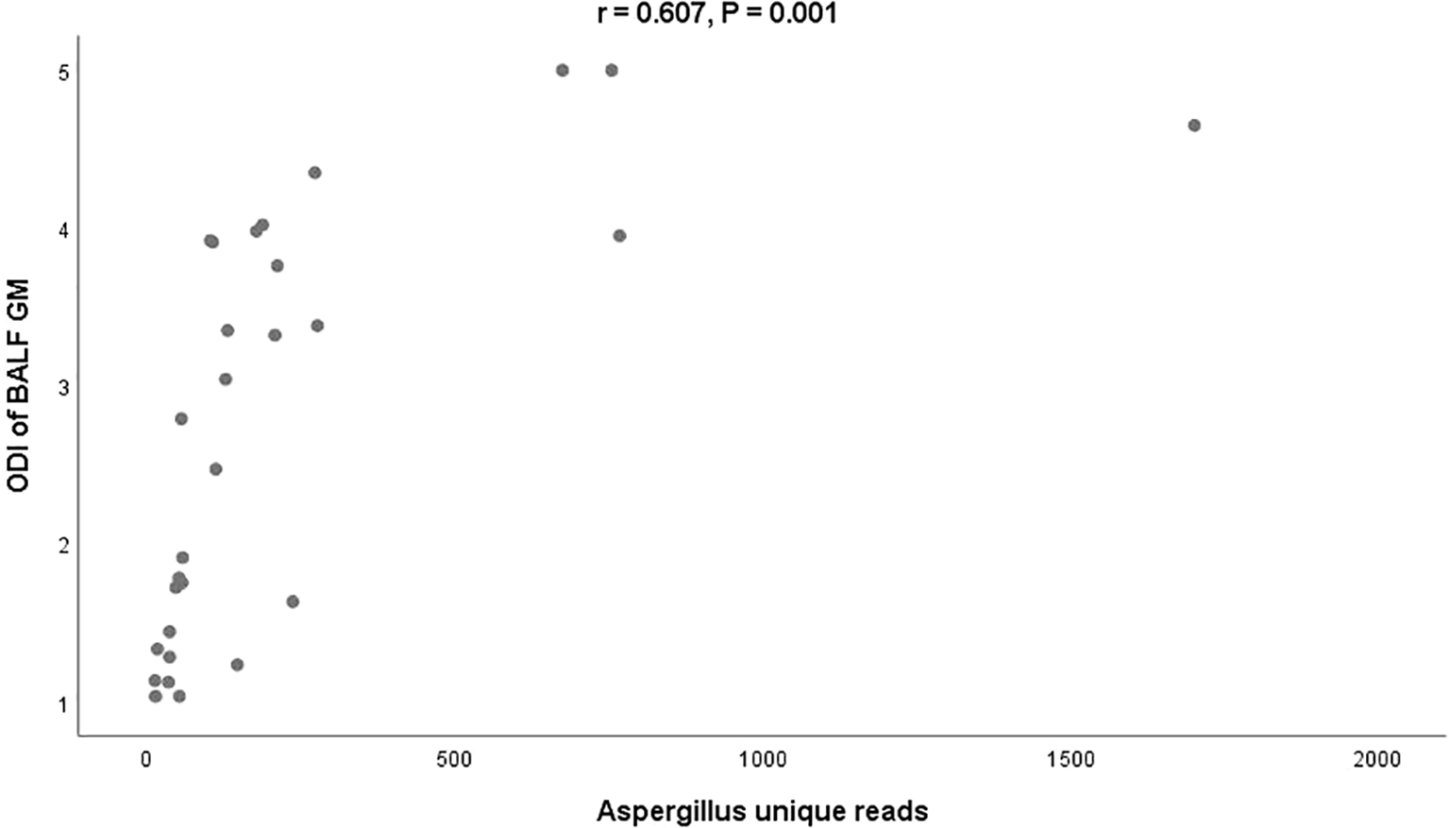
Relationship between the ODI of BALF-GM and Aspergillus unique reads by mNGS BALF, bronchoalveolar lavage fluid; GM, Galactomannan; mNGS, metagenomic next-generation sequencing; ODI, optical density index.

*Aspergillus* unique reads in cultured positive individuals were significantly higher than those in culture- negative individuals (131, IQR 82-222 vs. 56, IQR 47-107, *P* = 0.006). Although the unique reads of BALF-GM positive individuals were also higher than those of BALF-GM negative individuals, the difference was not statistically significant due to the small sample size of the BALF-GM negative group (107, IQR 51-205] vs. 48, IQR 40-92, *P* = 0.069). Additionally, a positive correlation was observed between the ODI of BALF GM and *Aspergillus* unique reads in BALF-GM positive patients (r = 0.607, *P* = 0.001) (**Figure 1**).

### Mixed infections and co-pathogens detected by mNGS in IPA patients

Thirty-two out of 37 patients (86%) with polymicrobial pneumonia were diagnosed by CMTs. However, when combined with mNGS results, the detection rate increased to 95% (*n* = 35). Pathogens missed by CMT included *Mucor* or *Rhizopus* spp. (*n* = 6), *P. jirovecii* (*n* = 3), influenza (*n* = 1), and nocardia (*n* = 1). The most common co-pathogen detected by mNGS was *P. jirovecii* (*n* = 17), followed by CMV (*n* = 13), bacteria (excluded *M.tuberculosis*) (*n* = 12), *Mucor* or *Rhizopus* (*n* = 7), influenza (*n* = 3) and *M. tuberculosis* (*n* = 3). The most common combination modes were *Aspergillus*-bacteria coinfection (*n* =12), *Aspergillus*-*P. jirovecii-*CMV coinfection (*n* = 9), and *Aspergillus*- *P. jirovecii* coinfection (*n* = 8) (**Supplementary Figure**).

## Discussion

The diagnosis of IPA is challenging due to the lack of characteristic clinical manifestations, making mycological evidence crucial for diagnosis. The slow growth of *Aspergillus* or its isolation under the lens or capsular bag can complicate pathogen detection (Bittinger et al., 2014; Clarke et al., 2018). Additionally, empirical antifungal therapy is increasingly common in high-risk groups of ICP, resulting in a low pathogen detection rate of CMTs (Perfect et al., 2001; Racil et al., 2011; Li et al., 2021). Therefore, there is a pressing need to explore new sensitive technology to improve *Aspergillus* identification.

mNGS has emerged as a promising tool for the diagnosis of infectious diseases, however, its clinical experience in diagnosing IPA is largely based on case reports and small case series. For instance, Yang et al. (Yang et al., 2022) and He et al. (He et al., 2019) reported cases of IPA in which mNGS quickly detected the pathogen, while other tests failed to identify it. Similarly, the largest study to date by Miao et al. (Miao et al., 2018) suggested that mNGS exhibited better performance than culture with regard to fungal detection, but the difference was not significant in *Aspergillus* detection (OR 3.7, 95% CI 0.97–20.5; *P* = 0.057) due to the small sample size of IPA (26 cases). Therefore, further validation by larger studies is necessary to confirm these preliminary findings.

In this study of 133 ICP, we foud that IPA was associated with severe clinical presentations and poor outcomes in critically ill patients. mNGS exhibited excellent performance in detecting Aspergillus. Its sensitivity outperformed any single CMT method, and can be comparable to the combination of multiple CMTs. Furthermore, mNGS performed well in identifying fungal species and co-pathogens, indicating that mNGS may have a valuable role in guiding antimicrobial therapy.

Upon reviewing relevant literature, it is noted that the vast majority of published studies only compared the performance of mNGS with a single CMT method for *aspergillosis* identification. For example, a series of studies found that the sensitivity of mNGS was better than that of culture (Chen et al., 2020; Bao et al., 2022); Additionally, the BALF GM test (range from 54.5% to 80%) outperformed serum GM (range from 24.3% to 46.9%) in the diagnosis sensitivity, which was widely reported and supported our results (Zhou et al., 2017; Dai et al., 2021; Ghazanfari et al., 2022). However, there have been few studies comparing the differences between BALF GM and mNGS. Ao et al. reported that BALF GM (57.7%) had the highest sensitivity in detecting *Aspergillus*, followed by mNGS (42.3%), culture (30.8%) and smear (7.7%) in 26 IPA patients (Ao et al., 2023). In contrast, our study observed improved sensitivity of mNGS compared to BALF GM. This divergence may be attributed to differences in the study population, sampling methods, empirical antifungal therapy, and even cutoff value (Stergiopoulou et al., 2007; Racil et al., 2011; Dai et al., 2021; Li et al., 2021). The aforementioned factors may reduce the sensitivity or increase false-positive of BALF GM (Cornillet et al., 2006; Pfeiffer et al., 2006; Stergiopoulou et al., 2007). Furthermore, the optimization of nucleic extraction methods from respiratory specimens may improve the ability of mNGS for *Aspergillus* identification (Zinter et al., 2019; Chen et al., 2020), which may explain the higher sensitivity of mNGS in this study. Notably, our study found a positive correlation between *Aspergillus* unique reads and fungal loads, and false-negative results of BALF GM or culture typically corresponded to smaller reads by mNGS, indicating that despite the difficulty of nucleic acid extraction, mNGS can still be a useful detection tool for low fungal load through technological improvements (Tsang et al., 2021; Jiang et al., 2022).

As is well known, accurate identification of strains is of great significance in guiding antifungal treatment. With the genomes of more than 1000 fungal species publicly available, mNGS offers a more accurate diagnostic analysis of pathogenic strains, which is even more specific than other methods (Chen et al., 2020; Etxebeste and Espeso, 2020; Yang et al., 2021). Our study found that only 39% of patients exhibited positive *Aspergillus* cultures, whereas mNGS not only identified strains that were highly consistent with those detected by culture, but also detected *Aspergillus* and even *Mucor* genera that were not detected by culture. Case reports highlighted the favorable prognosis resulting from antifungal drug changes based on mNGS results (Zhang et al., 2020; Yang et al., 2022). With an increase in high-risk populations receiving prophylactic antifungal therapy, there may be an increase in azole-resistant strains. However, mNGS has the potential to revolutionize the management of fungal infections by providing rapid and accurate identification of pathogenic strains, and even information on virulence and resistance to guide appropriate antifungal therapy (Diao et al., 2022; Jiang et al., 2022).

Our study also revealed that the vast majority of patients (80%) had mixed infections of *Aspergillus* with other pathogens, particularly “uncultivable” microorganisms such as CMV and *P. jirovecii*. Establishing an early diagnosis of co-infections remains a great challenge. CMT methods rely on pre-evaluating possible pathogens to carry out targeted detection. However, this approach is highly susceptible to the subjective experience of clinical personnel, and has a narrow pathogen spectrum, leading to a high rate of missed diagnosis. In contrast, mNGS offers an unbiased sampling approach, allowing for the simultaneous identification of all potentially infectious agents in a single run, without defining targets for diagnosis beforehand (Chen et al., 2022; Diao et al., 2022; Jiang et al., 2022). This may explain the satisfactory performance of mNGS in identifying co-pathogens in this study, which has also been confirmed by numerous other studies (Pan et al., 2019; Wang et al., 2019; Jiang et al., 2021). We believed that the mNGS strategy can not only simplify clinical testing for diagnosing mixed infections, but also bring the greatest benefits to ICP who often suffer from various complicated pathogen infection. Furthermore, due to the common co-pathogens being *P.jirovecii* and CMV, mNGS is more likely to reveal the etiology of infections when multiple PCR tests are rarely performed on respiratory samples.

Although our study has preliminarily demonstrated the feasibility of diagnosing IPA using mNGS, we must acknowledge the limitations of this technology. Firstly, the difficulty of nucleic acid extraction varied by species, and the number of unique reads was substantially reduced when contrasting fungi with bacteria pathogens (Fang et al., 2020; Jiang et al., 2022). Additionally, samples with high levels of human nucleic acid further reduced the identification of scarce microorganisms (Jacob, 2013; Grumaz et al., 2016). Furthermore, filamentous fungi mainly spread on the surface of lung tissue, making it difficult to wash pathogens off using lavage, resulting in small reads of fungi detected by BALF-mNGS (Yang et al., 2021). Several case reports have suggested that the reads numbers of *aspergillus* detected by mNGS as clinically significant pathogens range from 11 to 57 (He et al., 2019; Yang et al., 2022). Due to the inability to exclude low abundance organisms as potential causative agents (Li et al., 2018; He et al., 2019; Yang et al., 2022), the interpretation of the complex and diverse data generated by mNGS is more challenging. Even to this day, there is still a lack of widely accepted standards and quantitative thresholds (Miao et al., 2018; Fang et al., 2020; Qin et al., 2021; Yang et al., 2021; Ao et al., 2023). The evaluation for mNGS using absolute values as criteria like unique reads number remained controversial since the difficulty of extracting nuclei acids varies from species to species and the total sequencing yield varies from sample to sample (Li et al., 2018). Thus, mNGS cannot completely replace CMTs, but rather serves as a valuable adjunctive tool, particularly in scenarios where CMTs prove inadequate or yield inconclusive results. In addition, the interpretation of mNGS results requires advanced bioinformatics tools and expertise, as well as careful consideration of patient-specific factors, such as immune status, underlying diseases, antifungal pretreatment and clinical manifestations (Langelier et al., 2018; Diao et al., 2022; Jiang et al., 2022).

This study had several limitations that should be acknowledged. First, it was a single-center retrospective study; thus, intrinsic bias was unavoidable. Second, the diagnostic performances of mNGS and PCR method were not compared as PCR was not routinely performed in our hospital. This line of query should be explored in future work. Third, we used the clinical composite diagnostic criteria as the gold standard, but it must be acknowledged that even for experienced physicians, clinical misjudgment cannot be completely avoided, leading to deviations in diagnostic performance between different methods. Finally, the generalizability of mNGS is subject to certain limitations, and the next big challenges will be the optimization and standardization of sampling and mNGS analysis, which promise to enhance the clinical practicality of genomic diagnosis. Also, the potential benefits of mNGS in management of IPA warrant further investigation.

In summary, mNGS is feasible and highly sensitive method for detecting *Aspergillus* in ICP. It also performed well in identifying species of fungi and co-pathogens, providing significant benefits to ICP. However, due to the lack of widely accepted standards and inherent technical limitations, mNGS cann’t yet replace CMT, and the interpretation of its results should be combined with patient-specific factors.

## Data availability statement

The datasets presented in this article are not readily available because of license/restriction from the affiliated institution. Requests to access the datasets should be directed to YS, pumchshi@sina.com.

## Ethics statement

The studies involving humans were approved by The ethics committee of Peking Union Medical College Hospital. The studies were conducted in accordance with the local legislation and institutional requirements. The ethics committee/institutional review board waived the requirement of written informed consent for participation from the participants or the participants’ legal guardians/next of kin because given the retrospective design.

## Author contributions

YS conceived and designed this study. Material preparation and data collection by YS, J-MP and X-YH. Q-WY and YW was responsible for coordinating microbiological testing and interpretation. All authors were involved in drafting the article or revising it critically for important intellectual content. All authors contributed to the article and approved the submitted version.
