# Supplementary material for: Metagenomic next-generation sequencing for detecting Aspergillosis pneumonia in immunocompromised patients: a retrospective study
Source: Front Cell Infect Microbiol. 2023 Dec 22;13:1209724. doi: 10.3389/fcimb.2023.1209724 (PMC10770824; doi:10.3389/fcimb.2023.1209724)
Supplement: Supplementary file 1 [file Table_1.docx]

**Supplementary Table.** Detailed information of 46 patients with invasive pulmonary *aspergillosis*

| Patient No. | Underlying disease | Conventional Microbiological Test results | | | | BALF mNGS results | | | Final clinical Diagnosis | ICU Outcome |  |
| --- | --- | --- | --- | --- | --- | --- | --- | --- | --- | --- | --- |
|  |  | Fungal culture | Serum GM  (ODI) | BALF GM  (ODI) | Other | Fungi  (reads) | Bacteria | Viruses |  |  |  |
| 1 | ILD | *-* | - | 1.03 |  | *A. fumigatus* (53) |  |  | IPA | Alive |  |
| 2 | NHL | *-* | - | - | Culture: *P. aeruginosa;*  *A. baumannii* | *A.fumigatus (67)* | *P. aeruginosa*  *A. baumannii* | HHV-1 | IPA, *P. aeruginosa;*  *A. baumannii* | Death |  |
| 3 | ALL |  | - | 3.38 |  | *A.fumigatus* (277) |  | *-* | IPA | Alive |  |
| 4 | NPC | - | - | 2.79 | Culture: K. pneumonia | *A. fumigatus* (56) | K. pneumonia | - | IPA and K. pneumonia | Alive |  |
| 5 | ALL | *A. flavus* | - | 3.35 | Culture: K. pneumonia,  *A. baumannii* | *A. fumigatus* (131); *Rhizopus microsporus* (105) | K. pneumonia  *A. baumannii* | - | IPA; IPM; and  K. pneumonia;  *A. baumannii* | Alive |  |
| 6 | ILD | *-* | 0.88 | 0.91 | *-* | *-* |  | HHV-1 | IPA with ILD progression | Death |  |
| 7 | Testicular cancer | - | - | 3.76 | Culture: K. pneumonia,  *A. baumannii* | *A.fumigatus* (212) | K. pneumonia  *A. baumannii* | HHV-1 | IPA; K. pneumonia;  *A. baumannii* | Death |  |
| 8 | Wegener‘s granuloma | *A.fumigatus* | - | 1.72 | Culture: *A. baumannii;*  K. pneumonia;  qPCR for CMV (+) | *A.fumigatus* (47)  *A. flavus* (59) | *A.baumannii*  K. pneumonia | CMV | IPA, CMV and  *A. baumannii;*  K. pneumonia | Death |  |
| 9 | Gastric cancer |  | 0.73 | 0.90 | Culture: K. pneumonia | *-* | K. pneumonia;  C. striatum |  | IPA, C. striatum  K. pneumonia; | Alive |  |
| 10 | Renal carcinoma | *-* | - | 1.13 | qPCR for CMV(+) | *A．fumigatus (15)* |  | CMV | IPA and CMV | Death |  |
| 11 | SLE | *A.fumigatus* | - | 4.65 |  | *A. fumigatus* (1702)  *A. terrestris* (768)  *A. oryzae* (45) |  |  | IPA | Alive |  |
| 12 | Systemic vasculitis | *-* | - | 1.44 | *-* | *A. fumigatus* (37) |  |  | IPA | Alive |  |
| 13 | ILD | *-* | 0.78 | 0.84 | *-* | - |  |  | IPA with ILD progression | Death |  |
| 14 | SLE |  | - | 1.03 | *-* | *A. fumigatus* (14)  Pj (52) |  |  | IPA and PCP | Death |  |
| 15 | Systemic vasculitis | s | - | 1.32 | Culture: *P. maltophilia,*  *P. aeruginosa* | - | *P. maltophilia*  *P. aeruginosa* |  | IPA, *P. aeruginosa*  and *P. maltophilia* | Alive |  |
| 16 | SLE | *A.fumigatus* | - | 3.04 | qPCR for CMV(+)  PCR for Pj (+) | *A.fumigatus* (128)  *Mucor*spp (43)*；* |  | CMV | IPA, IPM, PCP and CMV | Death |  |
| 17 | Lymphoma | *-* | - | - | PCR for Pj (+) | *A. flavus*(16);  Pj (213) |  |  | IPA and PCP | Death |  |
| 18 | IPAF | *A.fumigatus* | - | - | PCR for Pj (+);  qPCR for CMV(+) | *A.fumigatus.* (58)*;*  Pj (444) |  | CMV | IPA, PCP and CMV | Death |  |
| 19 | NHL | *-* | 1.81 | 3.92 | Culture: *A. baumannii* | *A.fumigatus (103);*  *Rhizopus oryzae (49)* | *A. baumannii* | HHV-1  HHV-6 | IPA , IPM and  *A. baumannii* | Death |  |
| 20 | SLE | - | - | - | Anti-fast stain(+);  qPCR for CMV(+) | *A. fumigatus.* (15) | MTBC | - | Primary disease activity with IPA, CMV and M. *tuberculosis* | Death |  |
| 21 | Sjogren's syndrome | *A.fumigatusA. flavus* | 4.19 | 4.35 | PCR for Pj (+) | *A.fumigatus*(273)  *A.terrestris* (58)  Pj (121) |  |  | Primary disease activity with IPA and PCP | Death |  |
| 22 | Systemic vasculitis | *-* | - | 4.01 |  | *A.fumigatus*(188) |  |  | IPA | Alive |  |
| 23 | RA | - | 1.16 | 1.28 | GMS (+)；  PCR for Pj (+);  qPCR for CMV (+) | Pj (44)；  *A. fumigatus* (37) |  | CMV | IPA, CMV and PCP | Death |  |
| 24 | IIM | *-* | - | 3.95 | PCR for Pj (+) | *A.fumigatus* (768);  *Pj* (47) |  |  | IPA, PCP | Death |  |
| 25 | | Anti-Jo-1  syndrome | *A.fumigatus* | - | >5 | Influenza B PCR(+) | *A.fumigatus (755)*  *A. oryzae*(59) |  | Influenza B | IPA and *Influenza* B | Death |
| 26 | | Sjögren's  syndrome | - | - | 1.12 | Anti-fast stain(+) | *A. fumigatus* (35) | MTBC |  | IPA and *M. tuberculosis*; | Alive |
| 27 | | IIM |  | - | 1.02 | PCR for Pj (+)  qPCR for CMV (+) | Pj (447) |  | *CMV* | IPA, CMV and PCP | Alive |
| 28 | | IIM |  | 1.12 | - | qPCR for CMV (+) | Pj (136) |  | *CMV* | IPA , PCP and CMV | Death |
| 29 | | SLE | *A.fumigatusA. flavus*  *Rhizopus* | 1.99 | >5 | Culture: *A. baumannii* | *A.fumigatus (675)*  *A. flavus (234)*  *Rhizopus* spp (147) | *A. baumannii* |  | IPA, IPM and *A. baumannii ;* | Death |
| 30 | | AASV | *A.fumigatus* | 1.95 | - | qPCR for CMV(+) | *A. fumigatus (153);*  *A.terreus (53);*  *Pj (106)* |  | *CMV* | IPA*,* PCP and CMV | Death |
| 31 | | Systemic vasculitis | *A.fumigatus* |  |  | qPCR for CMV(+) | *A.fumigatus*. (59) |  | *CMV* | IPA, CMV | Death |
| 32 | | AASV | *A.fumigatus* | - | - | qPCR for CMV(+) | *A.fumigatus (41)* |  | *CMV* | IPA and CMV | Death |
| 33 | | AASV | *A.fumigatus* | 2.9 | 3.32 | PCR for Pj (+)  PCR for CMV (+) | *A.fumigatus (208);*  Pj (915) |  | CMV; HHV-1 | IPA, PCP, CMV | Death |
| 34 | | RA | *-* | - | 1.78 | PCR for influenza A (+) | *A.fumigatus* (52) |  | Influenza A*;*  *Coronavirus* 229E | IPA and influenza | Death |
| 35 | | SLE |  | 1.81 | 1.91 | culture *A.* *baumannii* | *A. terrestris*. (58)  *Rhizopus* spp (43) | *A. baumannii* | HHV-1 | IPA, IPM and  *A. baumannii;* | Death |
| 36 | | SLE | - | - | 2.47 |  | *A.fumigatus* (112) |  |  | IPA | Death |
| 37 | | AASV | *Aspergillus* spp | - | - | PCR (+) for *M. tuberculosis* | - | MTBC |  | IPA and  *M. tuberculosis* | Alive |
| 38 | | AML |  |  | 1.33 |  | *A.fumigatus* (17)  *A.oryzae* (11)；  *Rhizopus* spp (59) |  | Influenza B | IPA, IPM and Influenza B | Alive |
| 39 | | Nephrotic syndrome | *A.flavus*  *A.fumigatus* | 1.66 | 1.63 | PCR for Pj (+) | *A.fumigatus* (237)  *A.terrestris* (46)  Pj (56) |  |  | IPA and PCP | Death |
| 40 | | SLE | - | - | 1.13 | PCR for Pj (+)  qPCR for CMV(+) | Pj (17) |  | CMV | IPA ,PC P and CMV | Alive |
| 41 | | Lymphoma | *A.flavus* | 1.91 | 3.91 |  | *A.flavus* (107)  *A. niger* (23) |  |  | IPA | Alive |
| 42 | | ALL | - | - | 1.75 |  | *A.terreus*(57),  *Mucor*spp (31) |  |  | IPA and IPM | Death |
| 43 | | Nephrotic syndrome | *A. flavus* | - | - | GMS (+);PCR for Pj (+) | *A.flavus* (131),;  Pj (137) |  |  | IPA and PCP | Alive |
| 44 | | Lymphoma | *A.fumigatus* | - | 1.23 | PCR for Pj (+) | *A.fumigatus* (147);  *A.terreus* (37)，  *A. oryzae* (45)；  Pj (27) |  |  | IPA and PCP | Death |
| 45 | | Nephrotic syndrome | *A.flavus* | 1.33 | - | PCR (+) for Pj;  qPCR for CMV(+) | Pj (135)  *A.flavus (58)* |  | CMV | IPA, PCP and CMV | Death |
| 46 | | Lymphoma | - | - | 3.98 | GMS (+)；PCR (+) for Pj; | *A.flavus* (178);  Pj (345) | *Nocardia abscessus* |  | IPA, PCP and *Nocardia abscessus* | Death |

AASV: ANCA associated systemtc vasculitis; ALL: acute lymphoblastic leukemia; AML: acute myelocytic leukemia; BALF: bronchoalveolar lavage fluid; CMV: cytomegalovirus: GM: galactomannan; GMS: Gomori's Methenamine Silver; HHV: human herpes virus; ILD: interstitial lung disease; IIM: idiopathic inflammatory myositis; IPA: invasive pulmonary aspergillosis; IPAF: interstitial pneumonia with autoimmune features; IPM: invasive pulmonary mucormycosis; mNGS: metagenomic next-generation sequencing; MTBC: *Mycobacterium tuberculosis* complex; NHL: non-Hodgkin's lymphoma; NPC: nasopharyngeal carcinoma; ODI: optical density index; Pj: *P. jirovecii;* PCP: *P. jirovecii* pneumonia; PCR: polymerase-chain-reaction; qPCR: quantitative polymerase-chain-reaction; RA: rheumatoid arthritis; SLE: systemic lupus erythematosus.

A B

**Supplementary Figure.** Mixed infections and co-pathogens identified by mNGS in 46 patients with invasive pulmonary aspergillosis

A: number of IPA patients with mixed infections; B. number of IPA patients infected with various co-pathogens

CMV: cytomegalovirus; IPA: invasive pulmonary aspergillosis; PCP: *P. jirovecii* pneumonia; TB: *tuberculosis*
