# Supplementary material for: Metagenomic next-generation sequencing for detecting Aspergillosis pneumonia in immunocompromised patients: a retrospective study
Source: Front Cell Infect Microbiol. 2023 Dec 22;13:1209724. doi: 10.3389/fcimb.2023.1209724 (PMC10770824; doi:10.3389/fcimb.2023.1209724)
Supplement: Supplementary file 2 [file Table_2.docx]

**Appendix 1.** Detailed procedures of bronchoalveolar lavage fluid metagenomic next-generation sequencing

**Sample Acquisition, Processing, and Nucleic Acid Extraction**

Samples of 10 mL bronchoalveolar lavage fluid (BALF) were collected from patients according to standard procedures, and were immediately sent to the clinical laboratory. Initially, a 1.5 ml microcentrifuge tube (containing 0.5 ml BALF sample and 1 g 0.5-mm glass beads) was connected to a horizontal platform on the vortex mixer. Subsequently, the tube was agitated vigorously at 2,800–3,200 rpm for 30 min. Subsequently, the 0.3 ml sample was separated into a new 1.5 ml microcentrifuge tube and DNA/RNA was extracted using the TIA-Namp Micro DNA/RNA Kit (DP316, Tiangen Biotech Beijing, China) according to the manufacturer’s protocol.

**RNA enrichmen**

After mixing 33 microliters of the extracted nucleic acid sample with 7 microliters of the enrichment reaction mixture, incubate on a PCR machine at 37℃ for 10 min, and then perform magnetic bead purification to remove DNA from nucleic acids, thereby improving the concentration of RNA content.

**Reverse transcription and two-strand synthesis**

The unenriched nucleic acid or the enriched nucleic acid is subjected to fragmentation reaction, one-strand synthesis and two-strand synthesis to form double-stranded DNA nucleic acid, then purified by magnetic beads, and the purified DNA is used for DNA library construction.

**Construction of DNA library**

DNA libraries were constructed through an end-repair method in which the adapters were added overnight, adapter-ligation, and polymerase chain reaction (PCR) amplification was used prior to analysis using an Ion Torrent Proton Sequencer (Life Technologies, Carlsbad, California). The sequencing was performed on the BGISEQ-50/MGISEQ-2000 platform (Fang et al., 2018). The quality of the DNA libraries was assessed using an Agilent 2100 Bioanalyzer (Agilent Technologies, Santa Clara, California) combined with quantitative PCR to measure the adapters before sequencing. All sequencing reagents (PMSEQ) and PMseq software were approved by the China Drug Administration (CDA)

**Sequencing and Bioinformatic Analyses**

High-quality sequencing data (>10 million) were generated by removing low-quality and short-length (< 35 bp) reads (Schmieder and Edwards 2011), followed by a computational subtraction of human sequences mapped to the human reference genome (hg19) by Burrows–Wheeler alignment (Li and Durbin, 2010). Following completion of the above steps, the remaining sequence data were classified by simultaneously aligning with Pathogen Metagenomics Database (PMDB), consisting of bacteria, fungi, viruses, and parasites, which were downloaded from the National Center Biotechnology Information (<ftp://ftp.ncbi.nlm.nih.gov/genomes/>). It contains 4152 whole genome sequence of viral taxa, 3446 bacterial genomes or scaffolds, 206 fungi related to human infection, and 140 parasites associated with human diseases. The coverage ratio and the depth of each microorganism were calculated using BEDTools (Quinlan and Hall, 2010). The number of unique alignment reads was calculated and standardized to get the number of reads stringently mapped to pathogen species (SDSMRN) and the number of reads stringently mapped to pathogen genus (SDSMRNG) (Yao et al., 2016).

**Interpretation of metagenomic data and an official report to the clinician**

The microbial list obtained from the analysis process as described above, was compared to the background microbial database, an in-house database which contains microorganisms appearing in more than 50% of the samples in our laboratory in the past three months. Suspected background microorganisms were removed from the microbial list. This was determined based on the unique reads. Microorganisms with SDSMRN <50 unique reads but appearing at least 5 times or SDSMRN of >50 unique reads and appearing at least 3 times were considered as pathogens. For different types of microbes, the thresholds were set as follows (Wang et al., 2020; Doughty et al., 2014):

Bacterial/mycoplasma/chlamydia: SDSMRNG ≥3, if SDSMRN ≥3, species was reported; otherwise, the genus was reported.

DNA Virus/fungus: SDSMRN ≥3

RNA Virus: SDSMRN ≥1

Parasite: SDSMRN ≥100

Mycobacterium tuberculosis complex (MTC): SDSMRNG ≥1

Bacteria were listed in descending order of SDSMRNG, while viruses/fungi/parasites were listed in descending order of SDSMRN. The top 5 species were considered to be significant. The results were provided to the clinician in an official report and the clinician was required to identify pathogens based on the patient's clinical symptoms.

**Reference**

Doughty, E., Sergeant, M., Adetifa, I., Antonio, M., and Pallen, M. (2014). Cultureindependent

detection and characterisation of Mycobacterium tuberculosis and M. africanum in sputum samples using shotgun metagenomics on a benchtop sequencer. PeerJ. 2:e585. doi: 10.7717/peerj.585

Fang C, Zhong H, Lin Y, Chen B, Han M, Ren H, et al. 2018. Assessment of the cPAS-based BGISEQ-500 platform for metagenomic sequencing. Gigascience 7, 1-8. doi: 10.1093/gigascience/gix133

Li, H., and Durbin, R. (2010). Fast and accurate long-read alignment with Burrows-Wheeler transform. Bioinformatics 26, 589–595. doi: 10.1093/bioinformatics/btp698

Quinlan, A., and Hall, I. (2010). BEDTools: a flexible suite of utilities for comparing genomic features. Bioinformatics 26, 841–842. doi: 10.1093/bioinformatics/btq033

Schmieder, R., and Edwards, R. (2011). Quality control and preprocessing of metagenomic datasets. Bioinformatics 27, 863–864. doi: 10.1093/bioinfor matics/btr026

Wang H, Lu Z, Bao Y, Yang Y, Groot R de, Dai W, et al. Clinical diagnostic application of metagenomic next-generation sequencing in children with severe nonresponding pneumonia. PLoS ONE. (2020) 15:e0232610. doi: 10.1371/journal.pone.0232610

Yao M, Zhou J, Zhu Y, Zhang Y, Lv X, Sun R, et al. Detection of listeria monocytogenes in CSF from three patients with meningoencephalitis by next-generation sequencing. Journal of Clinical Neurology. 2016. October 1;12(4):446–51. 10.3988/jcn.2016.12.4.446
